# Fibula–Tibia Distance and Revision for Instability After Primary Total Knee Arthroplasty: An Exploratory Case–Control Study Addressing Rotational Confounding

**DOI:** 10.3390/jcm15197658

**Published:** 2026-10-02

**Authors:** Hamza Murad, Aia Bowirrat, Muhammad Khatib, Feras Qawasmi, Dror Robinson, Loai Abdallah, Mohammad Shehadeh, Hazem Safadi, Eitan Lavon, Mustafa Yassin, Assil Mahamid

**Affiliations:** 1 Department of Orthopaedic Surgery, Rabin Medical Center, Hasharon Hospital, Petah Tikva 4937211, Israelhazem.safadi@gmail.com (H.S.);; 2 Department of Information Systems, University of Haifa, Haifa 3498838, Israel; 3 Department of Information Systems, The Max Stern Yezreel Valley College, Emek Yezreel 1930600, Israel; 4 Management Department, Hasharon Hospital, Rabin Medical Center, Clalit Health Services, Petah Tikva 4937211, Israel

**Keywords:** total knee arthroplasty, instability, revision arthroplasty, radiographic morphometry, fibula, proximal tibiofibular joint, rotational alignment, case–control study

## Abstract

**Background/Objectives:** Instability without component loosening is a leading indication for revision total knee arthroplasty (TKA), yet no preoperative radiographic marker of this outcome has been validated. We examined whether preoperative fibula–tibia geometry on standing anteroposterior (AP) radiographs differs between patients subsequently revised for instability and non-revised controls, and whether any difference could be explained by radiographic rotation. **Methods:** In this exploratory retrospective case–control study, preoperative standing AP radiographs of 22 patients revised for instability without loosening (2014–2024) were compared with 29 primary-TKA controls frequency-matched on age, sex and comorbidity, analyzing the index (operated) knee in both groups. Three blinded orthopedic residents annotated 17 tibiofibular landmarks; 135 pairwise inter-landmark distances normalized to fibular head width were screened, and the distance with the best combined reliability–discrimination rank was carried forward with bootstrap optimism correction and nested repeated cross-validation. Four complementary rotation sensitivity analyses compared two rotation proxies between groups, adjusted the distance for them, decomposed it into vertical and horizontal components with an in silico rotation model, and assessed bilateral agreement. **Results:** The fibula lateral shaft–tibia lateral meta-diaphysis distance was shorter in revised patients (0.76 ± 0.16 vs. 0.93 ± 0.17 fibular-head-width units; *p* = 0.0006; area under the curve (AUC) 0.78 [95% CI 0.65–0.90], optimism-corrected 0.72, cross-validated 0.66; inter-rater ICC(A,1) 0.80). No statistically significant difference in either rotation proxy was detected, and the association persisted after adjustment for both proxies and side (odds ratio 4.00 per standard deviation, 95% CI 1.59–10.03). The difference resided mainly in the vertical component, which is less sensitive to simulated axial rotation under the model assumptions, and simulated rotation of 0–30° did not reproduce it. The bilateral agreement was moderate (r = 0.55), but the subsequently revised knee measured shorter than the contralateral knee. **Conclusions:** In this exploratory cohort a shorter fibula–tibia distance was associated with subsequent revision for instability, and the association persisted across the rotation sensitivity analyses performed. The distance is a hypothesis-generating candidate marker; it does not establish the risk of instability in unselected patients, and prospective, three-dimensional validation is required before any clinical use.

## 1. Introduction

Total knee arthroplasty (TKA) is among the most common orthopedic procedures, with demand projected to rise steeply [1]. Despite high satisfaction, a substantial minority of patients remain dissatisfied or require revision [2,3], and instability is one of the leading indications for reoperation—an etiology that contemporary institutional and registry series show to be stable or rising over time [4,5,6]. Flexion and global instability without component loosening are particularly difficult to diagnose and treat, and their definitions remain heterogeneous [7,8,9]. Management ranges from isolated polyethylene exchange to full component revision with escalating constraint, with outcomes dependent on careful patient selection [10,11,12,13].

While intraoperative soft-tissue balance and component position are recognized contributors, preoperative skeletal morphology may also predispose to instability [14,15]. Constitutional osseous variants—such as constitutional varus—are well established and, through contemporary coronal-plane phenotyping such as the Coronal Plane Alignment of the Knee classification, are increasingly considered in TKA planning [16,17,18]. The fibula anchors the principal posterolateral stabilizers, including the fibular collateral and popliteofibular ligaments, and forms the proximal tibiofibular articulation implicated in lateral-side restraint [19,20,21]. This anatomy makes the spatial relationship of the fibula to the lateral tibia a plausible candidate marker of lateral-side restraint; we emphasize, however, that none of these anatomical studies examined a radiographic fibula–tibia distance or its relationship to clinical instability, so the mechanistic link remains hypothetical. Prior radiographic work on instability has focused on postoperative parameters rather than preoperative constitutional geometry [22], even as image-based models have begun to predict arthroplasty-related outcomes from preoperative radiographs [23].

Using the AGSV (Annotation, Geometry, Statistics, Visualize) exploratory framework [24,25]—a landmark-based, inter-landmark distance-mapping approach of the kind increasingly applied to radiographic morphometry elsewhere in the musculoskeletal system [26]—we pursued two aims. The primary aim was to test whether preoperative fibula–tibia geometry on routine standing AP radiographs differs between patients subsequently revised for instability and non-revised primary-TKA controls; that is, to identify a candidate morphometric association with revision for instability, not to establish a validated predictor. The secondary aim was to assess whether any such difference could be attributed to radiographic rotation, because a coronal-plane distance measured on a two-dimensional radiograph can in principle be altered by knee rotation [27]. To this end, we subjected the candidate distance to a set of pre-planned rotation sensitivity analyses before proposing it as a marker.

## 2. Materials and Methods

### 2.1. Study Design and Population

This retrospective case–control study was approved by the Institutional Review Board of Rabin Medical Center, Hasharon Hospital, Petah Tikva, Israel (IRB number 0230-25-RMC; approved 18 August 2025), which waived informed consent for the use of anonymized radiographic data, and is reported according to the STROBE recommendations for case–control studies [28] (checklist in the Supplementary Materials). Human-rights and Declaration of Helsinki requirements were observed.

**Figure 1:**
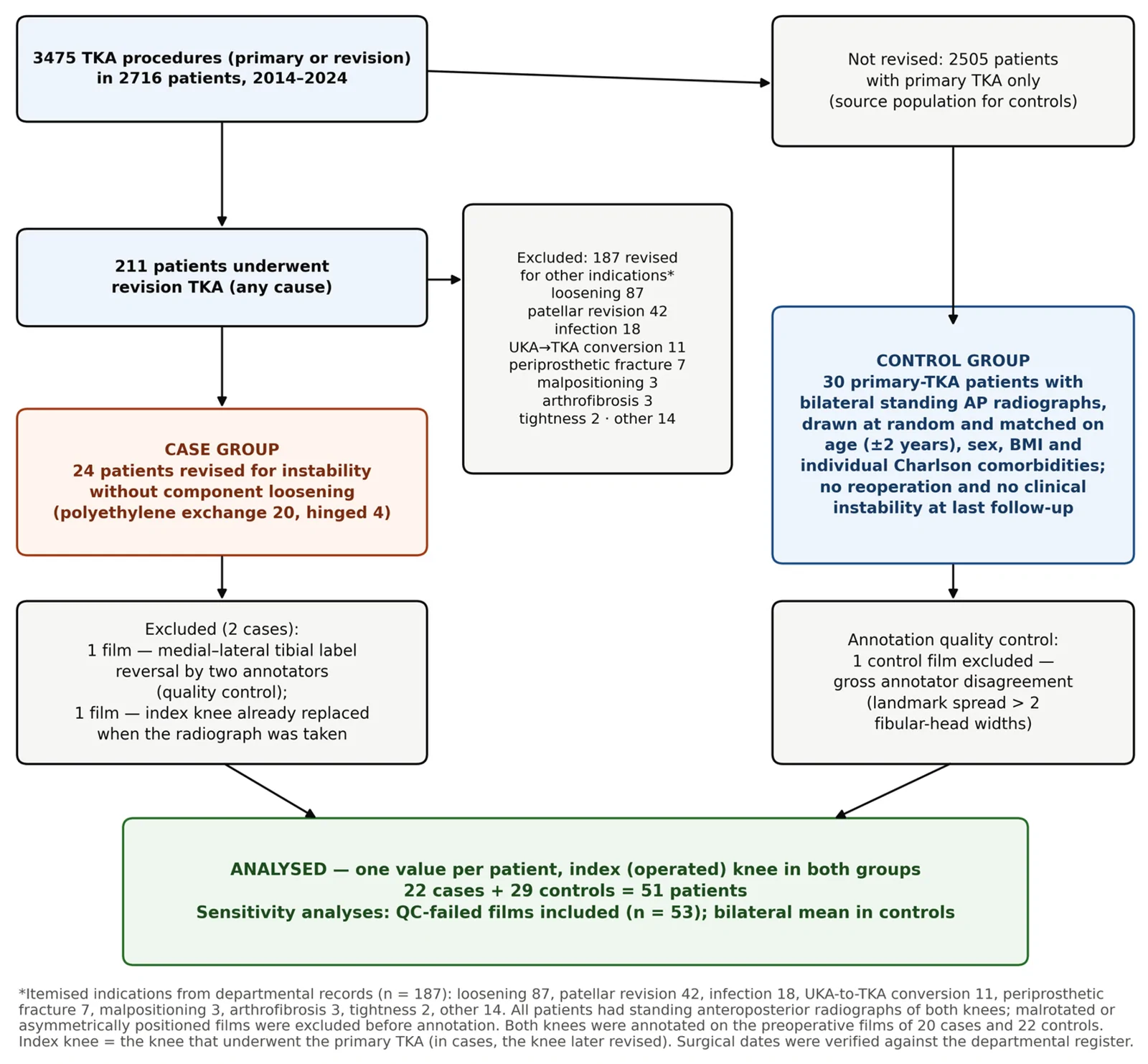
Patient flow. Of 3475 TKA procedures (primary or revision) in 2716 patients (2014–2024), 211 patients underwent revision TKA; 24 were revised for instability without component loosening (cases) and 187 for other indications (itemized from departmental records). Thirty primary-TKA controls were drawn at random from the 2505 non-revised patients and matched on age, sex, body mass index category and individual Charlson comorbidities. One case film and one control film failed the annotation quality control, and one further case was excluded because its annotated radiograph proved to show an already-replaced index knee, leaving 51 patients in the primary index-knee analysis; the sensitivity analysis including the films that failed quality control comprises 53 patients (23 cases, 30 controls).

Cases. The departmental surgical register of a primary and referral joint-surgery unit recorded 3475 TKA procedures (primary or revision) in 2716 patients between January 2014 and the data cut-off; 211 patients underwent revision TKA, and 24 of these had been revised specifically for instability without component loosening (Figure 1). Instability was diagnosed on the basis of anteroposterior laxity at 90° of flexion, mid-flexion laxity or varus–valgus opening on clinical examination and stress radiographs, and was confirmed by examination under anesthesia at the time of revision, as recorded in the operative notes. In the 24 source cases, the pattern documented in the operative notes was global instability in 21 patients—recorded as global, as instability in both extension and flexion, or as anteroposterior instability—and predominantly mediolateral instability in one, with recurvatum in two. Component loosening was excluded on preoperative radiographs and confirmed at surgery by direct assessment of component fixation, as recorded in the operative notes. Surgical management of the 24 source cases consisted of exchange to a thicker or more constrained polyethylene insert in 20 patients and conversion to a hinged prosthesis in four. Of the 24 source cases, the primary arthroplasty had been performed in the unit in 20 (register-verified dates); in four it had been performed before 2014 or at another institution, and details of the primary arthroplasty (implant design, constraint) were not systematically available.

Controls. Thirty controls were selected from the 2505 non-revised primary-TKA patients of the same unit recorded in the register (Figure 1) who had preoperative standing AP radiographs of both knees. Candidates were drawn at random from this source population and retained if they matched the case group on age (within ±2 years), sex, body mass index category (>30 kg/m^2^) and each individual comorbidity included in the Charlson comorbidity index, considered one by one rather than as a summary score; sampling stopped once a control group mirroring the cases on these characteristics had been assembled. The number of patients in the source population who met the radiographic criterion was not recorded, because candidates were screened only until the control group was complete. An uncomplicated outcome was defined as no reoperation, no clinical instability on examination, no complaint of giving way or recurrent effusion, and a satisfactory range of motion at the last follow-up visit. Freedom from reoperation was additionally verified against the departmental surgical register, which is complete to March 2025. No minimum follow-up was specified as an eligibility criterion; the documented clinical follow-up and the register-verified observation time of every patient are therefore reported explicitly (Section 3.1), and a sensitivity analysis restricted to controls with at least 24 months of documented observation is presented in Section 3.6. Because the case radiographs were necessarily older than the control radiographs (preoperative films of cases dated 2010–2023, of controls 2017–2025), the two groups were imaged during partly different calendar periods; the implications are considered in Section 4.

Radiographs and analytical unit. All patients had preoperative standing AP radiographs of both knees obtained with the departmental protocol (patellae facing forward, feet parallel, bilateral simultaneous exposure); films judged malrotated or asymmetrically positioned on visual review were excluded before annotation. The analytical unit was the index knee—the knee that underwent the primary TKA (in cases, the knee subsequently revised; in controls, the knee whose arthroplasty followed the annotated radiograph, or the first knee replaced in staged bilateral procedures, identified from the surgical register)—so that both groups contributed one value per patient from the same type of knee. Both knees were annotated on the preoperative films of 20 cases and 22 controls; these bilateral data were used only for the bilateral-agreement analysis and for a sensitivity analysis that averaged both knees in controls, as in the original submission. Two index films (one case, one control) failed the annotation quality control described below, and in one further case verification of the surgical dates against the register, with review of the image, showed that the annotated radiograph of the index knee had been obtained after that knee had already been replaced and was therefore not a preoperative film; these three patients were excluded, leaving 51 patients (22 cases, 29 controls) for the primary analysis (Figure 1). Unless stated otherwise, all descriptive statistics, percentages and results refer to these 51 patients; the source cohort of 54 patients (24 cases, 30 controls) is referred to only in the description of case ascertainment and in the sensitivity analyses (Section 3.6).

### 2.2. Landmark Annotation and Distance Mapping

**Figure 2:**
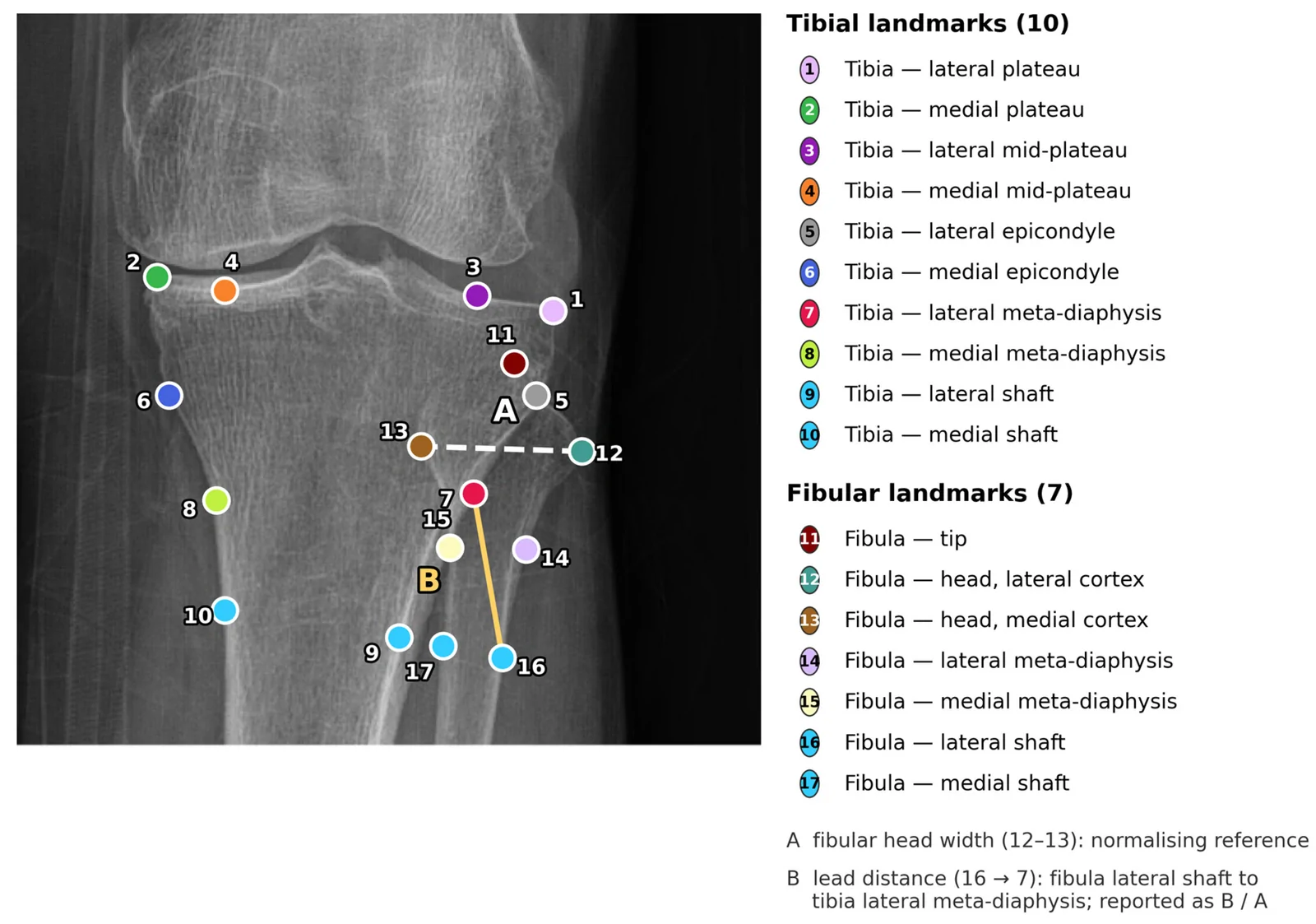
Annotated weight-bearing anteroposterior knee radiograph showing the 17 anatomical landmarks (numbered; 10 tibial, seven fibular) used for distance mapping. A (white dashed line), fibular head width (landmarks 12–13), the normalizing reference; B (yellow line), the lead distance from the fibula lateral shaft (16) to the tibia lateral meta-diaphysis (7). The reported standardized distance is B/A. Landmark definitions are given in Table S2.

A custom Python annotation application was used by three orthopedic residents (H.M., A.M., M.S.), who independently annotated 17 anatomical landmarks per knee (10 tibial, seven fibular; Figure 2). Landmark definitions are given in Table S2. The annotators were fully blinded to case–control status and to all clinical information and annotated independently using the written landmark definitions given in Table S2; no separate calibration session was held. Final coordinates were the per-landmark mean of the three annotators. All C(17,2) = 136 pairwise Euclidean inter-landmark distances were computed and normalized to the fibular head width—the distance between the medial and lateral cortices of the fibular head on the AP projection—to reduce the influence of radiographic magnification and of overall body size; the normalizing distance itself was excluded, leaving 135 distances. This reference was chosen because it lies within the structure of interest, is sharply defined on AP films, and does not depend on the tibial landmarks; it does not remove all body-size effects, and its projected width may itself vary with rotation and with individual tibiofibular morphology. Its distribution in the two groups and alternative scaling references are therefore examined explicitly (Section 2.4). Distances are reported in fibular-head-width units.

**Figure 3:**
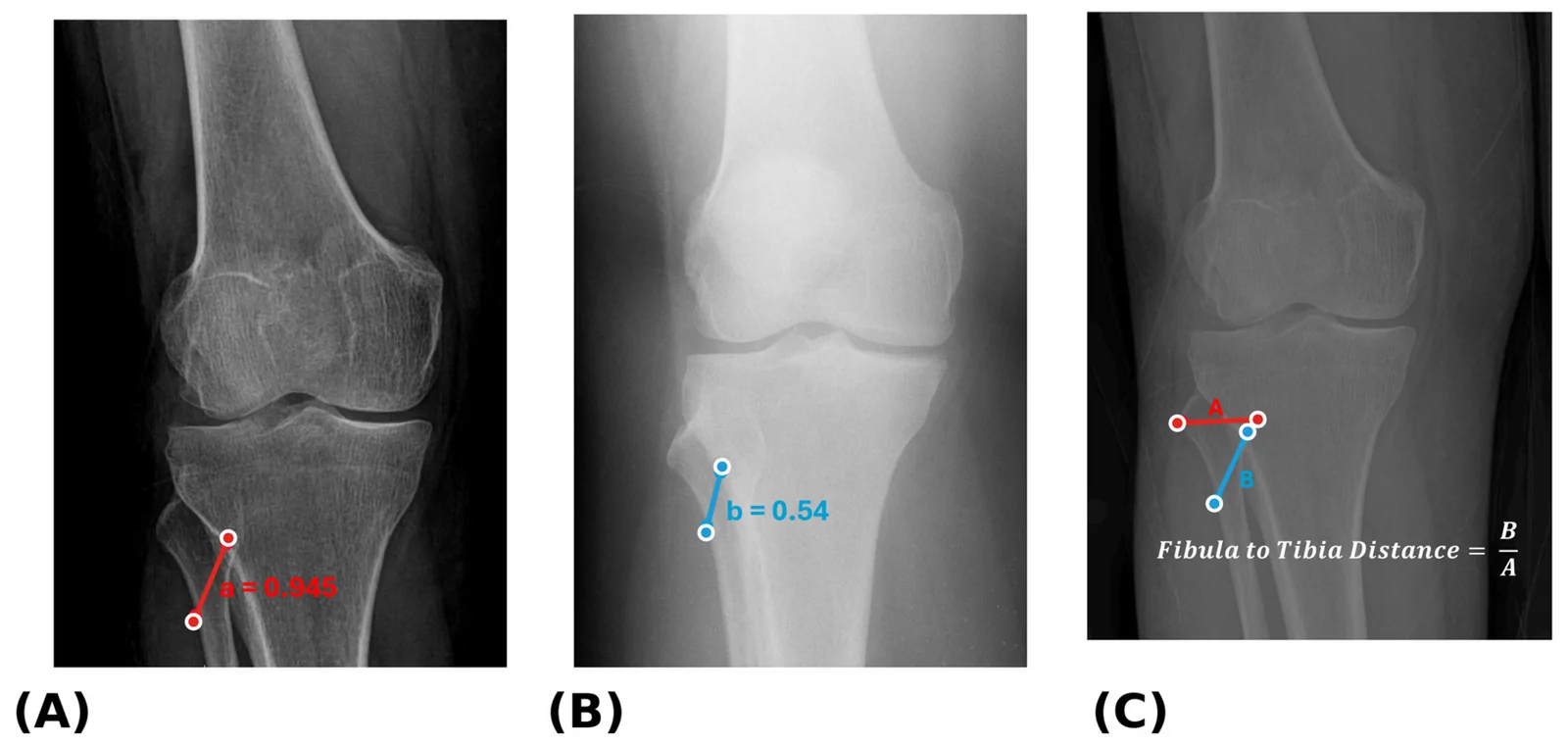
Representative standing anteroposterior radiographs illustrating the lead fibula–tibia distance (fibula lateral shaft–tibia lateral meta-diaphysis) and how it is measured; the two landmarks defining each measurement are marked. (**A**) Control knee, standardized distance 0.945—close to the control-group mean. (**B**) Revision-case knee, standardized distance 0.54—an illustrative example from the lower end of the revision-group distribution, chosen to make the measurement visible; the full distributions of both groups, which overlap substantially, are shown in Figure 4A. (**C**) Measurement method: line A spans the fibular head width (medial-to-lateral fibular head cortices), the normalizing reference; line B is the fibula lateral shaft–tibia lateral meta-diaphysis distance. The standardized distance reported throughout is the ratio B/A.

The distance carried forward, the fibula lateral shaft–tibia lateral meta-diaphysis distance, is the straight-line distance between (i) the lateral cortex of the fibular shaft at the level where the fibular neck ends and the shaft attains a parallel cortical outline, and (ii) the lateral cortex of the tibia at the meta-diaphyseal junction, where the flare of the lateral tibial metaphysis ends and the diaphyseal cortex becomes parallel (landmarks 16 and 7 in Figure 2 and Figure 3C).

Annotation quality control. Because the final coordinates are the mean of three annotators, a gross error by one annotator cannot be corrected by the others. An automated quality-control rule was therefore defined before the revised analyses were rerun and was applied to every film without knowledge of group status: a film was excluded if, for any annotator, a lateral tibial landmark (plateau or meta-diaphysis) lay on the non-fibular side of its medial counterpart (a medial–lateral labelling reversal), or if the between-annotator range of any landmark exceeded two fibular-head widths (gross misplacement). Four of the 117 annotated films (the index-knee films of all 54 source patients together with their contralateral-knee and repeat films) failed: one case index film (labelling reversal by two annotators), one control index film (gross disagreement) and two contralateral films of cases (labelling reversal), which affected only the bilateral analysis. All other films passed. The analysis including all films is reported as a sensitivity analysis.

**Figure 4:**
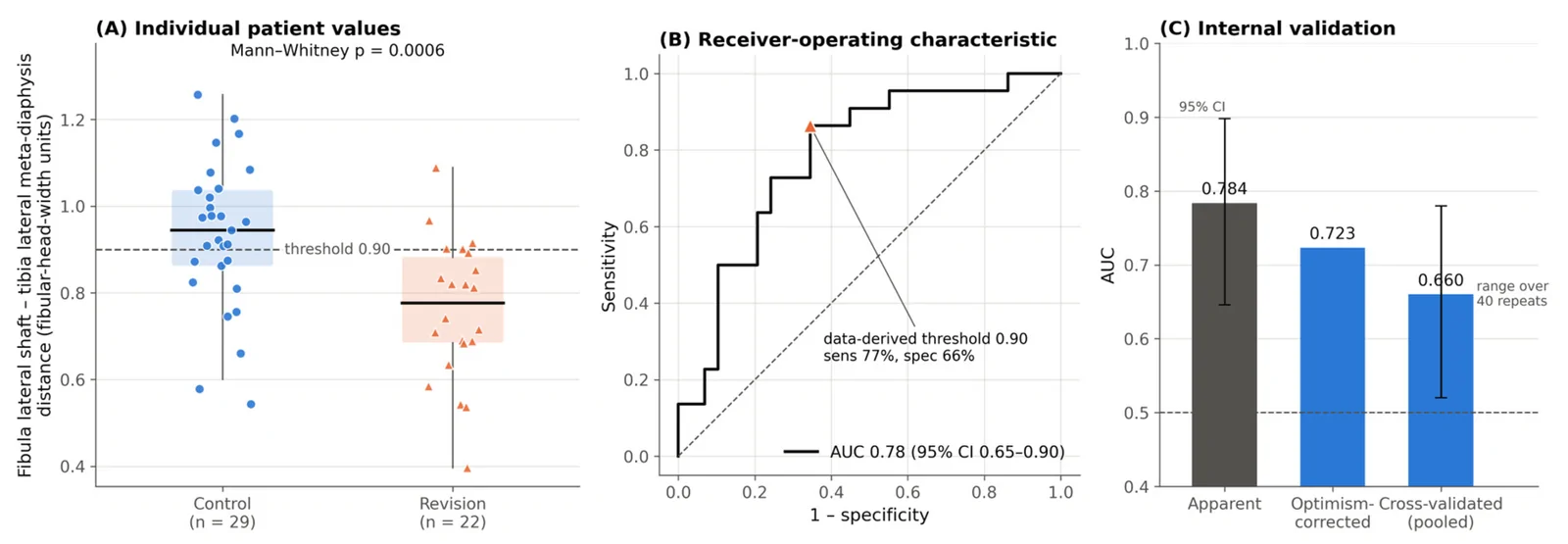
Lead distance in the primary index-knee cohort (n = 51). (**A**) Individual patient values with median, interquartile box and 1.5-IQR whiskers; the dashed line is the data-derived Youden threshold. (**B**) Receiver-operating-characteristic curve (the distance is inverted so that lower values indicate revision); the marker shows the data-derived threshold and the dashed diagonal indicates no discrimination. (**C**) Apparent, bootstrap optimism-corrected and cross-validated AUC; the error bar on the apparent AUC is the stratified bootstrap 95% CI and that on the cross-validated AUC the range over 40 repetitions of five-fold cross-validation with nested feature selection. The dashed horizontal line marks an AUC of 0.5.

### 2.3. Rotation Sensitivity Analyses

Four complementary sensitivity analyses were used to assess whether a case–control difference could be a rotation artefact. None can exclude rotation definitively; together they test whether the observed difference behaves as a rotation artefact would.

(i) Rotation proxies. Two indices of axial rotation were computed from the annotated coordinates of each film and compared between groups. Proxy 1, proximal tibiofibular overlap, was the mediolateral distance between the lateral margin of the tibial plateau and the medial cortex of the fibular head, divided by fibular head width; the projected overlap of the fibular head by the lateral tibia changes with axial rotation. Proxy 2, the lateral:medial tibial condylar width ratio, was the projected width of the lateral tibial condyle (lateral plateau margin to lateral mid-plateau point) divided by that of the medial condyle (medial plateau margin to medial mid-plateau point); rotation foreshortens the two condyles asymmetrically. Both are internally defined surrogates rather than validated rotation scales; their inter-rater reliability is reported alongside that of the distance.

(ii) Adjustment. The candidate distance was entered into a multivariable logistic model together with both proxies, and in a second model additionally with knee side, to test whether its association with revision persisted after accounting for measured positioning.

(iii) Decomposition and in silico rotation. The measured distance D was decomposed into a vertical component V (supero-inferior, along the limb axis) and a horizontal component H (mediolateral), D = (V^2^ + H^2^)^½, each normalized to fibular head width W. Axial rotation of the limb about its long axis leaves V unchanged in the AP projection but alters H. In a simplified geometric model, if the fibular shaft landmark lies at an antero-posterior offset d behind the tibial landmark, rotation by an angle θ changes the projected horizontal separation to H(θ) = |H·cosθ − d·sinθ|, and the projected normalized distance becomes D(θ) = (V^2^ + H(θ)^2^)^½/W(θ). Two variants of the denominator were evaluated: W(θ) = W (fibular head width unaffected by rotation, as in the original analysis) and W(θ) = W·cosθ (the fibular head width foreshortening with rotation, which is the more conservative assumption). The offset d is not measurable on AP films; on cross-sectional anatomy the fibular head lies roughly one head-width posterior to the lateral tibial cortex, so d = 1.0 W was used as the central value and d = 0.5 W and 1.5 W as sensitivity values. The model was run over θ = 0–30°, exceeding the malrotation of ±10–15° that can be expected on standardized standing films [27,29], using the mean control-group geometry (V, H, W). The question asked was whether any rotation in this range could drive the projected control-group distance down to the observed revision-group mean. The model represents rotation only about the limb axis and does not model knee flexion, beam divergence or three-dimensional tibiofibular anatomy.

(iv) Bilateral agreement. Within-patient agreement of the distance between the two knees was assessed in patients with bilateral films (Pearson correlation with 95% confidence interval [CI], two-way random-effects absolute-agreement intraclass correlation coefficient [ICC] and Bland–Altman limits of agreement [30]). In cases, the index knee was additionally compared with the contralateral knee.

### 2.4. Statistical Analysis

Distance screen. Each of the 135 normalized distances was compared between groups with the two-sided Mann–Whitney U test (α = 0.05). Multiplicity was addressed with the Bonferroni threshold (0.05/135 = 0.00037) and, less conservatively, the Benjamini–Hochberg false-discovery rate (FDR) [31]. For each distance, inter-rater reliability was quantified as the two-way random-effects absolute-agreement ICC for a single annotator, ICC(A,1), and for the mean of the three annotators, ICC(A,3), with 95% CIs computed with the formulae of McGraw and Wong [32], using the 77 quality-controlled films annotated by all three residents that comprised the index films of the 22 cases and all 55 films of the controls (the latter including the controls’ contralateral and repeat films, so that this set is not restricted to index knees); discrimination was quantified as the AUC (taken as the larger of AUC and 1 − AUC, so that it is direction-free). The candidate distance was selected by a combined rank: distances were ranked separately by AUC (descending) and by ICC(A,1) (descending), ties being assigned the minimum rank, and the distance with the lowest sum of the two ranks was carried forward (ties broken by AUC). This deliberately favours reliability over raw discrimination and does not necessarily select the distance with the smallest *p*-value. The full screen (group means, difference, *p*-value, FDR, AUC, ICC(A,1) and ICC(A,3) with CIs, and ranks) is provided in Table S1.

Lead distance. The between-group difference is reported as the Hodges–Lehmann shift with a bootstrap 95% CI and as the mean difference with CI. The AUC was accompanied by a stratified bootstrap 95% CI (2000 resamples). Because selecting the best of many distances inflates apparent performance, two internal-validation procedures were used, both repeating the entire selection procedure (all 135 distances) inside the resample or training fold so that no information from the held-out data entered the selection. (a) Bootstrap optimism correction: in each of 800 stratified bootstrap samples (seed 20260731) the selection was repeated, the AUC of the selected distance in the bootstrap sample was compared with its AUC in the original sample, and the mean optimism was subtracted from the apparent AUC [33]. (b) Repeated stratified five-fold cross-validation with 40 repetitions (seed 7): in each training fold the selection was repeated and the Youden threshold was determined, and the selected distance was scored on the held-out fold. The cross-validated AUC was computed by pooling the out-of-fold scores within each repetition (mean and range over the 40 repetitions), and the mean and standard deviation of the 200 fold-wise AUCs, the proportion of training folds in which the lead distance was selected, and the out-of-fold sensitivity and specificity at the fold-derived thresholds are also reported.

The classification threshold was derived from the present data as the Youden-optimal cut-off of the ROC curve; it was not pre-specified, and the sensitivity, specificity (Wilson 95% CIs) and odds ratio (OR; Haldane–Anscombe correction, Woolf CI) at this threshold are apparent sample estimates rather than validated performance measures. ORs per standard deviation of the distance were obtained from logistic regression, unadjusted and adjusted for the two rotation proxies and for knee side.

Sensitivity analyses. The primary analysis was repeated (a) including the films that failed quality control (53 patients: 23 cases and 30 controls; the case whose annotated film was not preoperative remained excluded); (b) using, as in the original submission, the mean of both knees in controls; (c) with the lead distance normalized to the tibial plateau width (medial to lateral plateau margins) instead of fibular head width, and unnormalized (pixels); and (d) stratified by knee side. The unnormalized fibular head width and tibial plateau width were compared between groups.

Missing data. All radiographic measurements were complete. The date of the last clinic visit, the date of the primary arthroplasty and the instability pattern were retrieved for every analyzed patient; the exact primary-arthroplasty date remains unavailable for one case operated at another institution before 2014, which is omitted from the interval summary only.

Analyses used Python 3.11.15 (Python Software Foundation, Wilmington, DE, USA) with NumPy 2.4.4, SciPy 1.17.1, scikit-learn 1.8.0 and pandas 3.0.2; ICC and logistic-regression estimators were implemented directly from the published formulae and verified against reference implementations. Analysis code is available from the corresponding author on request.

## 3. Results

### 3.1. Cohort

In total, 51 patients were analyzed (22 revision, 29 control; 88.2% female overall—86.4% and 89.7%, respectively; mean age 61.1 vs. 63.4 years). Baseline characteristics, follow-up and radiograph calendar periods are shown in Table 1. Smoking was recorded in 6 of 22 (27.3%) revision patients and 2 of 29 (6.9%) controls (OR 4.3, 95% CI 0.9–21.1); no other characteristic differed appreciably, although with 22 cases the CIs of all baseline comparisons are wide and the absence of a significant difference should not be read as evidence of equivalence. The median interval from primary TKA to revision for instability was 21 months (IQR 18–27; range 8–58). Clinical follow-up after the index arthroplasty was shorter in controls than in cases (14 months, IQR 6–33, vs. 46 months, IQR 25–56; *p* = 0.0002), as expected when patients doing well attend less often; controls were nevertheless observed in the surgical register for a median of 26 months (IQR 21–71) after the index arthroplasty without any recorded reoperation. Eight of the 29 controls had less than 24 months of documented observation, i.e., less than the median interval at which cases were revised; a sensitivity analysis restricted to the remaining controls is reported in Section 3.6. The annotated radiograph preceded the index TKA by a median of 0.6 months in controls and 0.4 months in cases, but by more than two years in two cases, in whom the contralateral knee had been replaced first or surgery had been deferred. The control index knee was the right knee in 16 and the left knee in 13 controls; in cases it was the right knee in 10 and the left in 12.

### 3.2. Candidate Distance and Internal Validation

Of the 135 normalized distances, 51 differed between groups at *p* < 0.05, 15 met the Bonferroni threshold and 37 met an FDR of 5% (Table S1); as in the original analysis, most of the distances that differed connected the fibula to the lateral tibia and were shorter in the revision group (Figure S1). The fibula lateral shaft–tibia lateral meta-diaphysis distance had the best combined rank (AUC rank 18 of 135, ICC(A,1) rank 30, combined rank 1) and was carried forward; its exact *p*-value was 0.0006 (which does not meet the Bonferroni threshold of 0.00037; FDR-adjusted *p* = 0.0041).

The lead distance was shorter in revision patients: mean 0.76 ± 0.16 (median 0.78, IQR 0.68–0.88) vs. 0.93 ± 0.17 (median 0.94, IQR 0.86–1.04) fibular-head-width units; Hodges–Lehmann difference −0.17 (95% CI −0.27 to −0.08); mean difference −0.17 (95% CI −0.26 to −0.08). The individual values overlap considerably (Figure 4A). The apparent AUC was 0.78 (95% CI 0.65–0.90); the optimism-corrected AUC was 0.72 (mean optimism 0.061) and the cross-validated AUC 0.66 (range over 40 repetitions 0.52–0.78; fold-wise mean 0.68 ± 0.18) (Figure 4B,C). The lead distance was re-selected in only 56% of the 200 training folds; in most of the remaining folds a neighbouring fibula-to-lateral-tibia distance (fibula lateral shaft–tibia lateral shaft or fibula lateral meta-diaphysis–tibia lateral meta-diaphysis) was selected instead. The cross-validated AUC therefore describes the out-of-sample performance of the whole selection procedure rather than of the lead distance alone, and is lower than the optimism-corrected AUC of the lead distance itself. The instability of the selection among closely related distances is itself informative: the signal appears to be carried by a family of fibula-to-lateral-tibia distances rather than by one uniquely privileged measurement. The out-of-fold sensitivity and specificity at fold-derived thresholds were 68% and 55%.

The Youden-optimal, data-derived threshold was 0.90 fibular-head-width units (Figure 4B). At this threshold, sensitivity was 77% (95% CI 57–90%) and specificity 66% (95% CI 47–80%); patients below the threshold had higher odds of belonging to the revision group (OR 5.9, 95% CI 1.7–20.0; Fisher *p* = 0.004). Per standard deviation, a lower distance carried an unadjusted OR of 3.17 (95% CI 1.48–6.83). Performance is summarized in Table 2.

### 3.3. Inter-Rater Reliability

Inter-rater reliability of the lead distance across the 77 quality-controlled films (22 case index films and 55 control films, including the controls’ contralateral and repeat films; Section 2.4) was ICC(A,1) = 0.80 (95% CI 0.65–0.88) for a single annotator and ICC(A,3) = 0.92 (95% CI 0.85–0.96) for the mean of three annotators, the quantity used in the analysis. Pairwise Bland–Altman analysis showed small systematic offsets between annotators (bias −0.03 to +0.10 units) with limits of agreement of about ±0.18 units (Figure S2). Reliability of the fibular head width was high (ICC(A,1) 0.94), of rotation proxy 1 high (ICC(A,1) 0.93), and of rotation proxy 2 only moderate (ICC(A,1) 0.51, 95% CI 0.38–0.63), which limits the weight that can be placed on the latter.

### 3.4. Rotation Sensitivity Analyses

**Figure 5:**
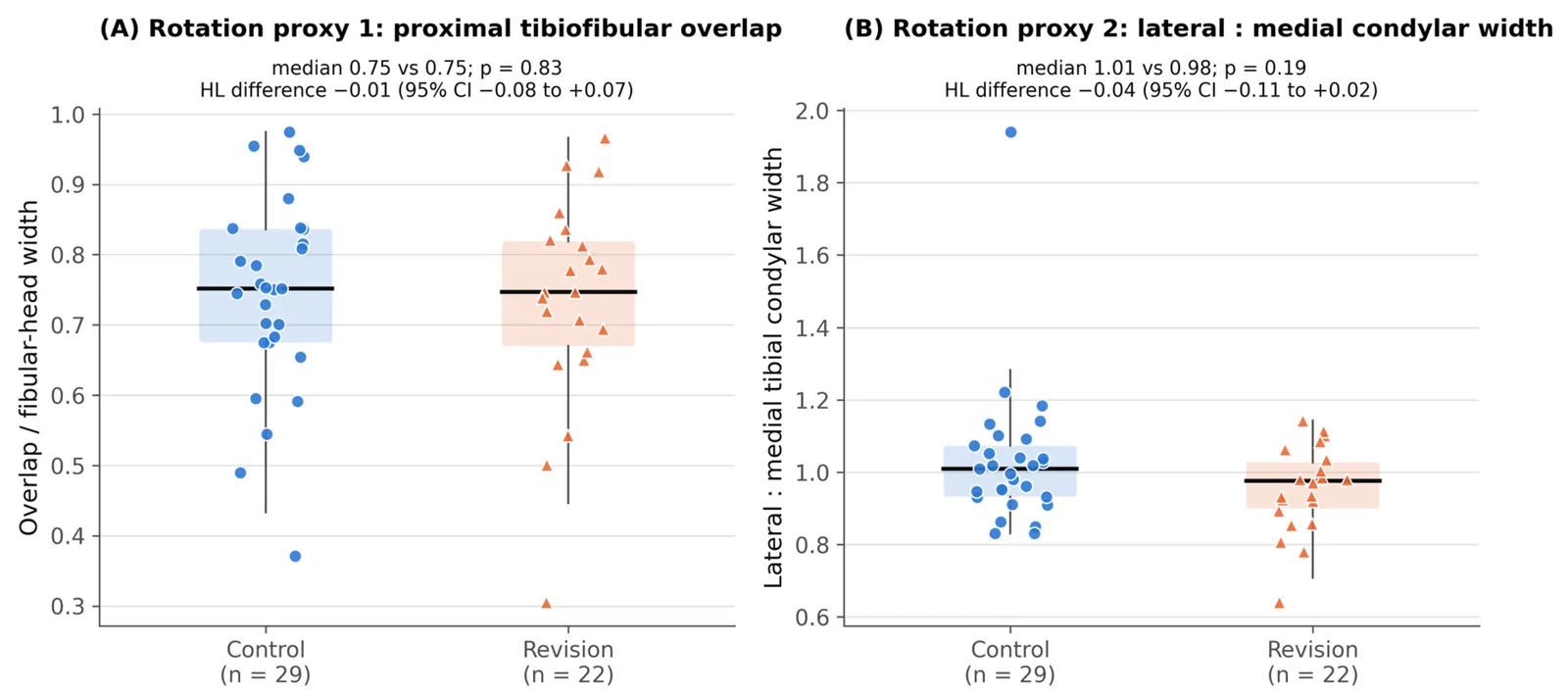
The two radiographic rotation proxies in the primary cohort (n = 51): (**A**) proximal tibiofibular overlap (inter-rater ICC(A,1) 0.93); (**B**) lateral:medial tibial condylar width ratio (ICC(A,1) 0.51). Points are individual patients; boxes show median and interquartile range. HL, Hodges–Lehmann estimate of the revision-minus-control difference.

No statistically significant between-group difference was detected in either rotation proxy: proximal tibiofibular overlap, median 0.75 vs. 0.75 (Hodges–Lehmann difference −0.01, 95% CI −0.08 to +0.07; *p* = 0.83); condylar width ratio, median 1.01 vs. 0.98 (difference −0.04, 95% CI −0.11 to +0.02; *p* = 0.19) (Figure 5). These CIs are compatible only with small positioning differences for proxy 1 but, given the moderate reliability of proxy 2, are less informative for the latter. After adjustment for both proxies, the association of the distance with revision persisted (adjusted OR 3.61 per SD, 95% CI 1.56–8.36, *p* = 0.003), and it persisted with knee side added to the model (OR 4.00, 95% CI 1.59–10.03, *p* = 0.003); neither proxy nor side was itself associated with group.

**Figure 6:**
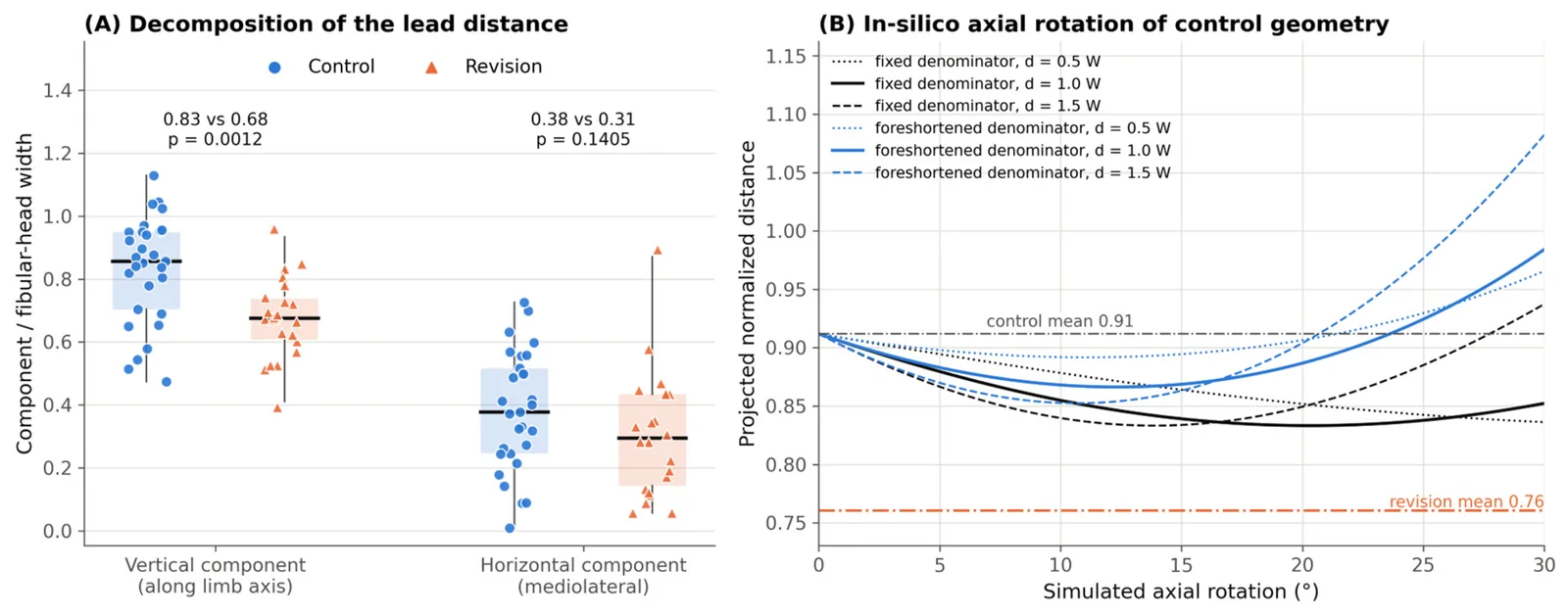
Rotation sensitivity of the lead distance. (**A**) Decomposition into the vertical component (along the limb axis, unchanged by axial rotation in the model) and the horizontal component (mediolateral, rotation-sensitive), each normalized to fibular head width. (**B**) In silico axial rotation of the mean control-group geometry over 0–30° for three assumed antero-posterior fibular offsets (d = 0.5, 1.0 and 1.5 fibular-head widths, W), with the fibular head width either fixed (black) or foreshortening with rotation (blue). The projected distance does not reach the revision-group mean under any of the assumptions examined. The model represents rotation about the limb axis only.

Decomposition showed that the between-group difference resided mainly in the vertical component (V/W 0.83 ± 0.17 vs. 0.68 ± 0.13; *p* = 0.001), which lies along the axis of rotation and is therefore less sensitive to simulated axial rotation under the model assumptions, whereas the rotation-sensitive horizontal component differed less (H/W 0.38 ± 0.19 vs. 0.31 ± 0.20; *p* = 0.14) (Figure 6A). In the in silico model, rotating the mean control geometry through 0–30° reduced the projected normalized distance from 0.91 to a minimum of 0.83 with the fixed denominator (d = 1.0 W; 0.83–0.84 across d = 0.5–1.5 W) and to 0.87 with the foreshortening denominator (0.85–0.89); the revision-group mean of 0.76 was not reached under any combination of assumptions examined (Figure 6B). The rotation-related results are collated in Table 3.

### 3.5. Bilateral Agreement

**Figure 7:**
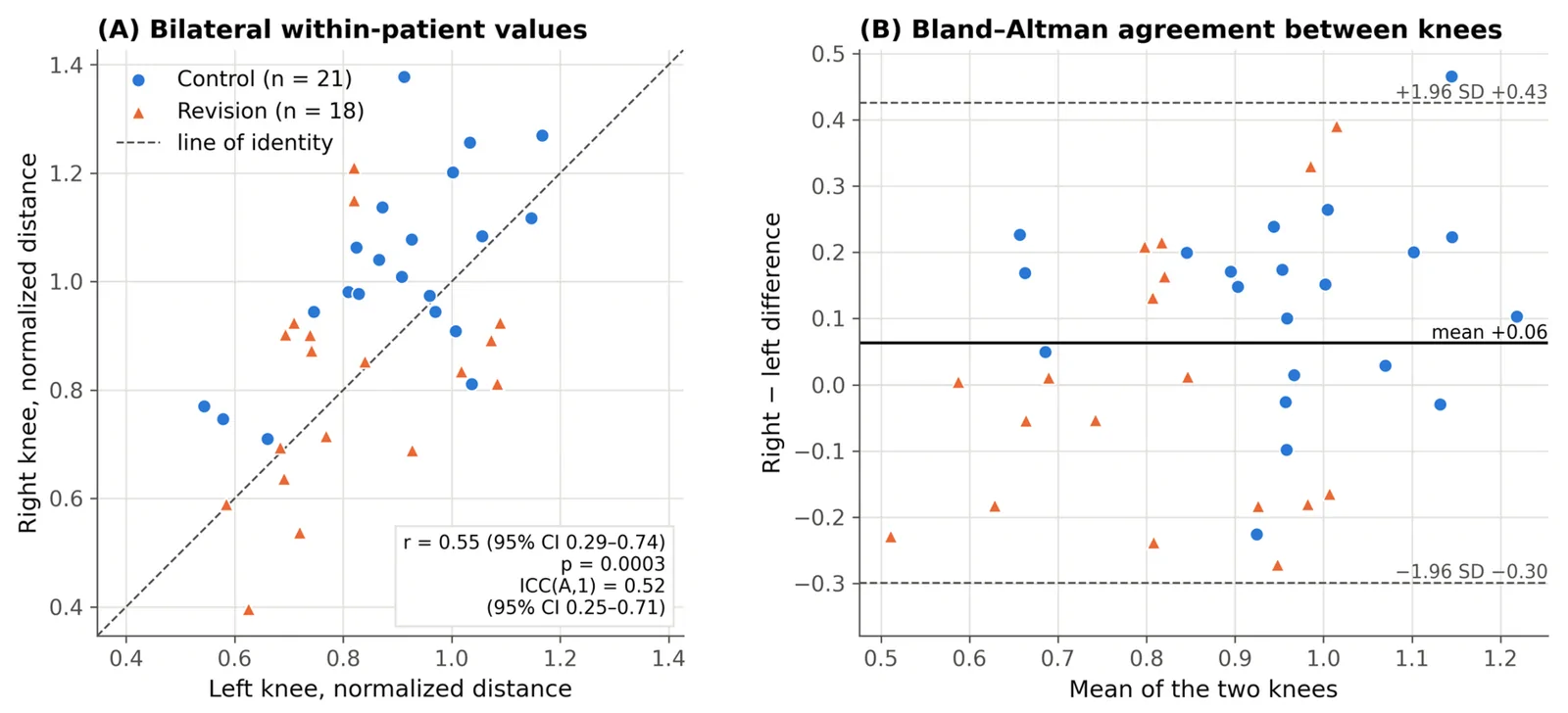
Bilateral within-patient agreement of the normalized fibula–tibia distance in 39 patients with bilateral films. (**A**) Right versus left knee with the line of identity; r, Pearson correlation; ICC, two-way random-effects absolute-agreement intraclass correlation. (**B**) Bland–Altman plot of the right-minus-left difference against the mean of the two knees, with the mean difference and 95% limits of agreement.

Among 39 patients with bilateral preoperative films that passed quality control (18 cases, 21 controls), the distance of the two knees was correlated (Pearson r = 0.55, 95% CI 0.29–0.74; *p* = 0.0003; Spearman ρ = 0.52) with an ICC(A,1) between knees of 0.52 (95% CI 0.25–0.71); the Bland–Altman mean right-minus-left difference was +0.06 (limits of agreement −0.30 to +0.43) (Figure 7). Two observations qualify the interpretation of this symmetry. First, the right knee measured systematically higher than the left within bilateral films of controls (mean difference +0.12, *p* = 0.002), a positioning- or annotation-related laterality effect for which side was therefore included as a covariate (Section 3.4). Second, in the 18 cases the subsequently revised knee measured shorter than the patient’s contralateral knee (0.76 vs. 0.86; Wilcoxon *p* = 0.03), and the bilateral correlation was weaker in cases (r = 0.38) than in controls (r = 0.63). The distance therefore showed moderate within-person similarity together with a knee-specific component; the bilateral data cannot separate constitutional anatomy from shared positioning and do not provide firm support for a constitutional interpretation, which remains a hypothesis.

### 3.6. Sensitivity Analyses

Sensitivity analyses are summarized in Table 4. Including the two films that failed quality control (n = 53; 23 cases, 30 controls) attenuated the difference (0.81 vs. 0.92; *p* = 0.005; AUC 0.73), because the affected case film carried an artefactually high value and the affected control film an artefactually low one; the direction and significance of the association were unchanged. Using the mean of both knees in controls, as in the original submission, gave 0.76 vs. 0.94 (*p* = 0.0002; AUC 0.81). The unnormalized fibular head width was, if anything, smaller in cases (184 ± 35 vs. 205 ± 24 pixels; *p* = 0.04), so normalization to it works against rather than for the observed difference; the unnormalized lead distance was also shorter in cases (140 ± 40 vs. 191 ± 38 pixels; *p* < 0.0001; AUC 0.83), although pixel values are confounded by magnification and detector differences, and normalization to tibial plateau width gave an AUC of 0.77 (*p* = 0.001). Within each side the association was in the same direction (left knees: 0.77 vs. 0.86, AUC 0.65; right knees: 0.75 vs. 0.99, AUC 0.93). Restricting the control group to the 21 controls with at least 24 months of documented observation, so that controls with the least opportunity to declare instability were removed, gave 0.76 vs. 0.93 (*p* = 0.001; AUC 0.79).

## 4. Discussion

The principal finding of this exploratory case–control study is that a single fibula–tibia distance measurable on routine preoperative standing AP radiographs was shorter in patients later revised for instability than in non-revised controls, with an association that survived internal validation (optimism-corrected AUC 0.72) and that was consistent across the four rotation sensitivity analyses performed: no significant difference was detected in either of two internally defined rotation proxies (one of which had only moderate inter-rater reliability), the association persisted after adjustment for both proxies and knee side, the discriminating signal lay predominantly in the vertical component, which is less sensitive to simulated axial rotation under the model assumptions, and simulated rotation of up to 30° could not reproduce the observed difference. Because even modest axial malrotation can measurably alter projected coronal relationships on plain radiographs [29], such convergent analyses are necessary before a two-dimensional coronal measurement can be proposed as a morphological marker. They reduce the likelihood that rotation explains the association; they do not exclude positioning effects definitively, and in particular they do not address knee flexion, beam angulation, landmark-placement error or three-dimensional tibiofibular anatomy, which only cross-sectional imaging can resolve [34].

The anatomical rationale is plausible but untested. The fibula anchors the posterolateral corner [19,20,35], and a fibula sited closer to the lateral tibia might reflect a configuration in which the principal lateral restraints act at a shorter effective lever. We hypothesize that, if such a configuration were constitutional—by analogy with constitutional varus [16,17] and with the wide distribution of native coronal phenotypes [36]—it could persist after arthroplasty because it resides in bone that resurfacing does not alter. None of the present radiographic or clinical data test this mechanism, and the cited anatomical and biomechanical studies do not examine a radiographic fibula–tibia distance or its relationship to instability. The bilateral analysis does not provide firm support for a constitutional interpretation: the subsequently revised knee measured shorter than the same patient’s contralateral knee, and bilateral agreement, although present, was only moderate. The distance may therefore combine a constitutional component with a knee-specific component (for example deformity- or positioning-related), which the present design cannot separate. This extends prior radiographic work, which has emphasized postoperative measures [22], by identifying a preoperative candidate that the AGSV distance-mapping framework [24,25] is well suited to surface, but the mechanism remains a hypothesis for prospective and cross-sectional study.

Several features of the design constrain interpretation. The outcome was revision for instability, not instability itself: revision depends on symptom severity, diagnostic practice, referral patterns, surgical indications and access to care, so the distance is associated with a clinical decision as well as with a biological state, and patients with unrecognized or unrevised instability may be present among controls despite their documented follow-up. Odds ratios from a case–control sample in which cases are enriched relative to the source population (24 cases among 2716 patients) do not represent the absolute or relative risk of instability in unselected patients undergoing primary TKA, and the threshold-based estimates are data-derived and unvalidated. The cases were also imaged, on average, several years earlier than the controls, and referral of instability cases from other institutions (four of the 24 source cases had undergone their primary arthroplasty before 2014 or at another institution) introduces the possibility of referral and surveillance bias as well as period-related differences in radiographic technique and implant use. Follow-up was also asymmetric: cases necessarily remained under observation until revision and beyond, whereas controls, who were doing well, attended less often (median clinical follow-up 46 months in cases versus 14 months in controls, *p* = 0.0002). Controls were nevertheless observed in the surgical register for a median of 26 months, longer than the median interval of 21 months at which cases were revised, and the association was unchanged when controls with less than 24 months of documented observation were excluded (Section 3.6); a control who developed instability after the last recorded contact, or who was revised at another institution, would nonetheless have been misclassified. Non-differential misclassification of this kind could attenuate the association, whereas misclassification related to the measurement could bias it in either direction, and the sensitivity analysis restricted to controls with at least 24 months of observation does not fully address the follow-up imbalance or the possibility of revision at another institution. Smoking was substantially more frequent among cases (OR 4.3, 95% CI 0.9–21.1) even though the comparison did not reach conventional significance, and preoperative coronal alignment, deformity severity, ligament status, implant design (available only for the revision procedure), surgical technique, component position and rotation, gap balancing and surgeon were not available for adjustment; with 22 events, extensive multivariable adjustment was not feasible in any case. The association reported here should therefore be read as one that persisted after adjustment for the two measured rotation proxies and side, not as independence from all confounding. The predominantly female cohort limits generalizability to men.

Our estimates are deliberately conservative. Analyzing one index knee per patient, correcting for selection optimism by bootstrap and by cross-validation with nested selection, as recommended for small-sample prediction problems [33], and excluding films with objectively identified annotation errors under a rule applied uniformly to both groups, yielded an optimism-corrected AUC of about 0.72 and an OR at threshold of about 6; the cross-validated performance of the complete selection procedure was lower still (AUC 0.66), because closely related fibula-to-lateral-tibia distances compete for selection in small training folds, and the analysis including all films, in which the two artefactual values happened to act in opposite directions, is reported so that readers can judge the effect of that exclusion. We frame the distance as a candidate morphometric marker and proof of concept, not as a validated predictor.

### Limitations

This is a single-centre, retrospective case–control study with a modest sample; it is underpowered for extensive multivariable modelling, and the adjusted estimates, while significant, have wide confidence limits. Control selection, follow-up and the calendar-period difference between groups are described transparently but could not be fully controlled; in particular, the number of source-population patients screened before the control group was complete was not recorded, so selection bias in assembling this small control group cannot be excluded. The rotation proxies are internally defined surrogates rather than validated indices, the condylar width ratio in particular had only moderate inter-rater reliability, and the in silico model is a simplified geometric representation that requires an assumed fibular antero-posterior offset and a fixed or cosine-foreshortened reference width; its conclusions apply to axial rotation of 0–30° under those assumptions. The normalizing fibular head width is anatomically related to the structure under study; its distribution did not favour the finding, and alternative scaling gave similar results, but no two-dimensional reference is free of projection effects. A systematic right–left difference within bilateral films indicates side-dependent projection or annotation effects that were adjusted for but not explained. In two cases the annotated radiograph preceded the arthroplasty by more than two years, in one by eleven years, because the contralateral knee was replaced first or surgery was deferred; the geometry of an arthritic knee may change over such an interval. Plain radiography cannot fully exclude out-of-plane rotation, and three-dimensional CT—already used to characterize the proximal tibiofibular relationship in three dimensions [34]—would permit rotation-independent measurement and is the natural next validation step. Finally, the marker is exploratory: like any candidate prediction rule, it requires prospective, multicentre and external—ideally three-dimensional—validation before any clinical application [37].

## 5. Conclusions

In this exploratory case–control study, a shorter preoperative fibula lateral shaft–tibia lateral meta-diaphysis distance on standing AP radiographs was associated with subsequent revision for instability after primary TKA. The measurement showed acceptable inter-rater reliability in the present sample, the association persisted after internal validation and across the rotation sensitivity analyses performed, and it was not reproduced by simulated axial rotation within the range examined. The distance is a hypothesis-generating candidate marker whose mechanism has not been tested; it does not establish the risk of instability in unselected patients, and prospective, three-dimensional validation is required before any clinical use.

## Figures and Tables

**Table 1:** Baseline characteristics of the primary analysis cohort (*n* = 51).

Characteristic	Revision (*n* = 22)	Control (*n* = 29)	*p*
Age, years, mean ± SD	61.1 ± 9.0	63.4 ± 9.1	0.47
Female, n (%)	19 (86.4%)	26 (89.7%)	1.00
Current smoking, n (%)	6 (27.3%)	2 (6.9%)	0.06
BMI > 30 kg/m^2^, n (%) ‡	17 (77.3%)	22 (75.9%)	1.00
Charlson comorbidity index, mean ± SD	1.4 ± 1.3	1.4 ± 1.4	0.86
Diabetes mellitus, n (%)	8 (36.4%)	15 (51.7%)	0.39
Cardiovascular disease, n (%) §	9 (40.9%)	9 (31.0%)	0.56
Any malignancy, n (%)	3 (13.6%)	4 (13.8%)	1.00
Index knee = right, n (%)	10 (45.5%)	16 (55.2%)	0.58
Year of preoperative radiograph, median (range)	2021 (2010–2023)	2022 (2017–2025)	—
Interval from annotated radiograph to index TKA, months, median (range)	0.4 (0.0–137)	0.6 (0.0–21)	—
Interval from primary TKA to revision for instability, months, median (IQR) ¶	21 (18–27)	—	—
Clinical follow-up after index TKA, months, median (IQR) ‖	46 (25–56)	14 (6–33)	0.0002
Observation in the surgical register after index TKA, months, median (IQR) #	—	26 (21–71)	—

Values are mean ± SD, median (interquartile range) or n (%). Categorical variables compared with Fisher’s exact test, continuous variables with the Mann–Whitney U test. ‡ BMI was recorded categorically (>30 kg/m^2^) in the source records. § Cardiovascular disease = myocardial infarction, congestive heart failure, peripheral vascular disease, or cerebrovascular disease. The Charlson comorbidity index was derived from documented comorbidities (age component excluded). ¶ Available for 21 of 22 cases; the exact primary-arthroplasty date is unavailable for the remainder, operated before 2014 or at another institution. ‖ Time from the index TKA to the last documented clinic visit. # Time from the index TKA to the register cut-off (March 2025) without any recorded reoperation at the institution.

**Table 2:** Performance of the lead distance (fibula lateral shaft–tibia lateral meta-diaphysis), primary index-knee cohort (n = 51).

Metric	Estimate	95% CI
Revision vs. control, mean ± SD (fibular-head-width units)	0.76 ± 0.16 vs. 0.93 ± 0.17	—
Hodges–Lehmann difference (revision − control)	−0.17	−0.27 to −0.08
Mann–Whitney U test	*p* = 0.0006	—
Inter-rater reliability, single annotator, ICC(A,1)	0.80	0.65–0.88
Inter-rater reliability, mean of three annotators, ICC(A,3)	0.92	0.85–0.96
AUC, apparent	0.78	0.65–0.90 (bootstrap)
AUC, optimism-corrected (800 bootstraps, selection repeated)	0.72	—
AUC, cross-validated (5-fold × 40, nested selection)	0.66	range 0.52–0.78
Data-derived (Youden) threshold	0.90	—
Sensitivity/specificity at threshold (apparent)	77%/66%	57–90%/47–80%
Sensitivity/specificity, cross-validated	68%/55%	—
Odds ratio, distance below threshold	5.9	1.7–20.0
Odds ratio per SD lower, unadjusted	3.17	1.48–6.83
Odds ratio per SD lower, adjusted for rotation proxies and side	4.00	1.59–10.03

AUC, area under the receiver-operating-characteristic curve; CI, confidence interval; ICC, intraclass correlation coefficient (two-way random effects, absolute agreement); OR, odds ratio; SD, standard deviation. The threshold, sensitivity, specificity and threshold-based OR are data-derived apparent estimates.

**Table 3:** Rotation sensitivity analyses (primary index-knee cohort, n = 51).

Analysis	Control	Revision	Estimate (95% CI)	*p*
Proxy 1: proximal tibiofibular overlap, median	0.75	0.75	HL −0.01 (−0.08 to +0.07)	0.83
Proxy 2: lateral:medial condylar width ratio, median	1.01	0.98	HL −0.04 (−0.11 to +0.02)	0.19
Distance, OR per SD lower, adjusted for both proxies	—	—	3.61 (1.56–8.36)	0.003
Distance, OR per SD lower, adjusted for proxies and side	—	—	4.00 (1.59–10.03)	0.003
Vertical component V/W, mean ± SD	0.83 ± 0.17	0.68 ± 0.13	—	0.001
Horizontal component H/W, mean ± SD	0.38 ± 0.19	0.31 ± 0.20	—	0.14
In silico rotation 0–30°, fixed denominator: minimum projected distance (d = 0.5–1.5 W)	0.91 (0°)	0.76 (mean)	floor 0.83–0.84	—
In silico rotation 0–30°, foreshortening denominator: minimum projected distance	0.91 (0°)	0.76 (mean)	floor 0.85–0.89	—
Bilateral agreement, Pearson r (n = 39)	—	—	0.55 (0.29–0.74)	0.0003
Bilateral agreement, ICC(A,1) between knees	—	—	0.52 (0.25–0.71)	—
Cases: index vs. contralateral knee, mean (n = 18)	—	0.76 vs. 0.86	—	0.03

CI, confidence interval; HL, Hodges–Lehmann estimate (revision minus control); ICC, intraclass correlation coefficient; OR, odds ratio; SD, standard deviation; W, fibular head width. Values in fibular-head-width units unless stated.

**Table 4:** Sensitivity analyses of the lead distance.

Analysis	n (rev/ctrl)	Revision, Mean ± SD	Control, Mean ± SD	*p*	AUC
Primary: index knee, QC-passed films	22/29	0.76 ± 0.16	0.93 ± 0.17	0.0006	0.78
All films, no QC exclusion	23/30	0.81 ± 0.27	0.92 ± 0.18	0.005	0.73
Mean of both knees in controls (original analytical unit)	22/30	0.76 ± 0.16	0.94 ± 0.14	0.0002	0.81
Normalized to tibial plateau width	22/29	0.27 ± 0.05	0.33 ± 0.06	0.001	0.77
Unnormalized lead distance (pixels)	22/29	140 ± 40	191 ± 38	<0.0001	0.83
Fibular head width, unnormalized (pixels)	22/29	184 ± 35	205 ± 24	0.04	—
Left index knees only	12/13	0.77	0.86	0.22	0.65
Right index knees only	10/16	0.75	0.99	0.0004	0.93
Controls with ≥24 months of documented observation	22/21	0.76 ± 0.16	0.93 ± 0.15	0.001	0.79

AUC, area under the curve; QC, annotation quality control. *p*-values from the Mann–Whitney U test. Pixel values depend on magnification and detector and are not comparable across films; they are shown only to demonstrate that the finding is not created by the normalization. In the all-films analysis, the case whose annotated film was not preoperative remains excluded (23 cases); in the bilateral-mean analysis, the control whose index film failed quality control contributes its remaining quality-controlled film (30 controls).

## Data Availability

The complete per-distance results of the screen (Table S1) are provided as Supplementary Material. The underlying landmark coordinates, radiographs and clinical data are not publicly available because they contain patient identifiers and radiographic information that could compromise the privacy of the patients included; de-identified measurement data and the analysis code are available from the corresponding author on reasonable request, subject to institutional review board approval.

## Supplementary Materials

The following supporting information can be downloaded at: https://www.mdpi.com/article/10.3390/jcm15197658/s1, Figure S1: Exploratory all-knee distance screen from the original submission—inter-landmark distances differing significantly between cohorts (unadjusted *p* < 0.05), overlaid on a representative knee; Figure S2: Inter-rater agreement for the lead distance (fibula lateral shaft–tibia lateral meta-diaphysis); Table S1: Screen of all 135 normalized inter-landmark distances in the primary index-knee cohort; Table S2: Anatomical definitions of the 17 landmarks annotated on the standing anteroposterior radiograph; STROBE Statement—checklist of items for reports of case–control studies.
