# Supplementary material for: Fibula–Tibia Distance and Revision for Instability After Primary Total Knee Arthroplasty: An Exploratory Case–Control Study Addressing Rotational Confounding
Source: J Clin Med. 2026 Oct 2;15(19):7658. doi: 10.3390/jcm15197658 (PMC13666231; doi:10.3390/jcm15197658)
Supplement: Supplementary file 1 [file jcm-15-07658-s001.zip › Supplementary_Materials_JCM_R2.pdf]

Supplementary Materials

Fibula–Tibia Distance and Revision for Instability After Primary Total Knee Arthroplasty: An Exploratory Case–Control Study Addressing Rotational Confounding

Murad H, Bowirrat A, Khatib M, Qawasmi F, Robinson D, Abdallah L, Shehadeh M, Safadi H, Lavon E, Yassin M, Mahamid A. Journal of Clinical Medicine, manuscript jcm-4556235 (revision 1).

Contents: Figure S1; Figure S2; Table S1 (provided as a separate Excel workbook, Table_S1_all_135_distances.xlsx); Table S2; STROBE checklist (separate file).

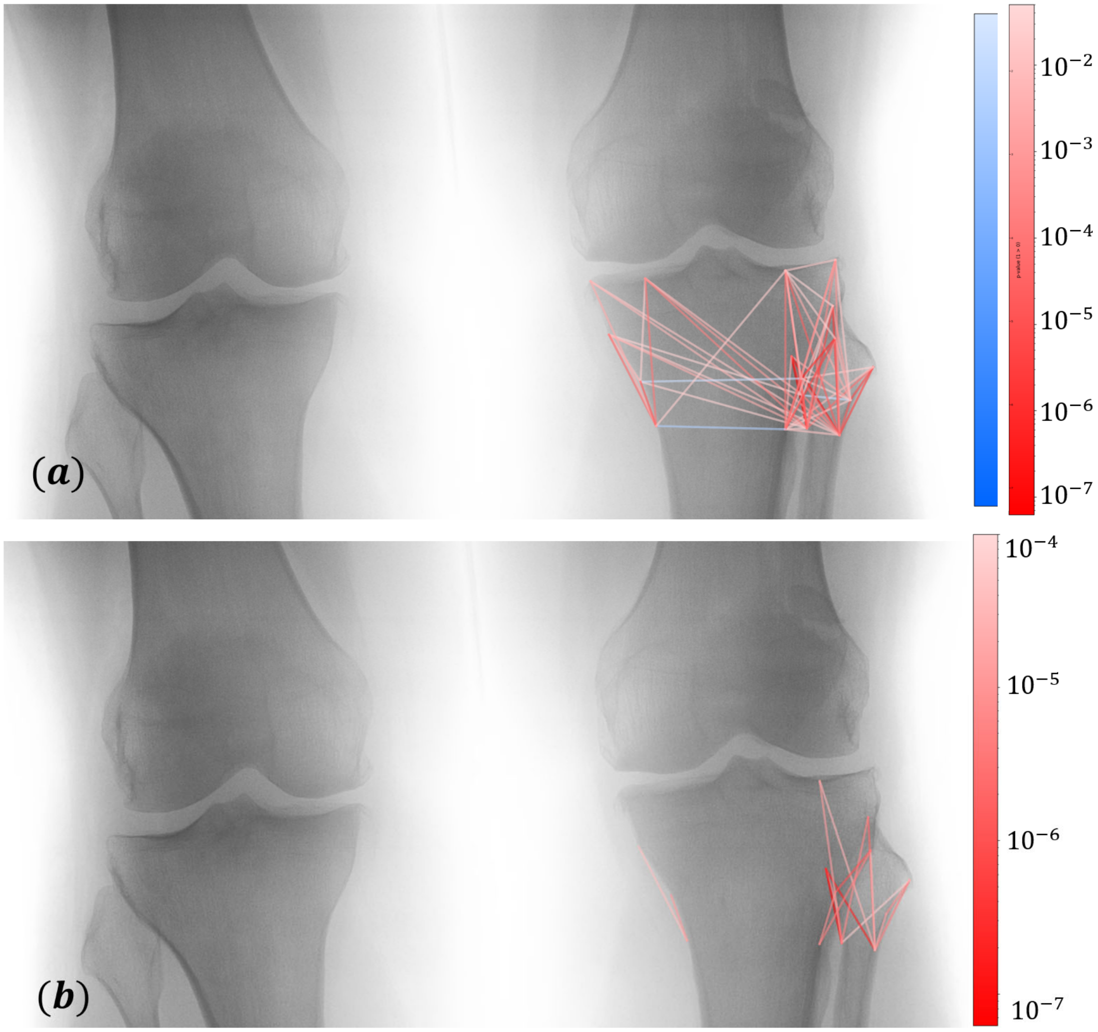

Figure S1. Exploratory all-knee distance screen from the original submission—inter-landmark distances differing significantly between cohorts (unadjusted $p < 0.05$), overlaid on a

representative knee. Blue, shorter in the revision group; red, longer. This figure is retained for orientation; the quantitative screen on the primary index-knee cohort is given in Table S1.

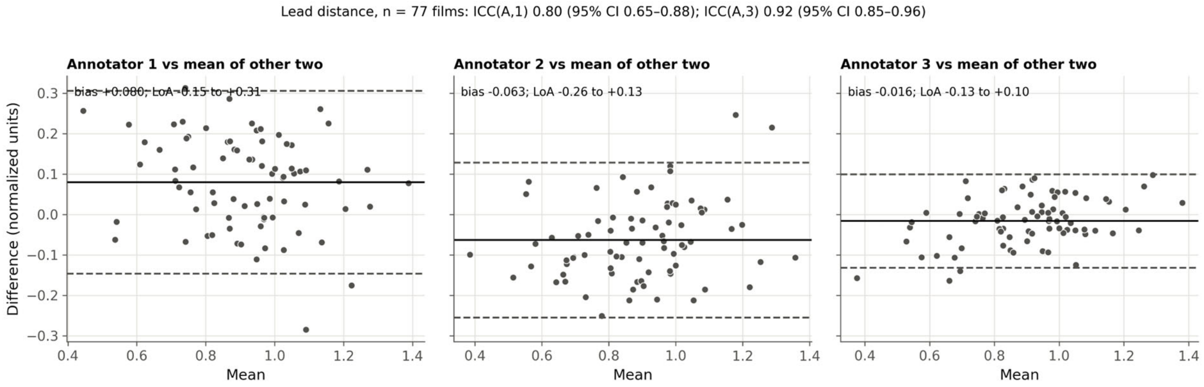

Figure S2. Inter-rater agreement for the lead distance (fibula lateral shaft–tibia lateral meta-diaphysis, fibular-head-width units) across the 77 quality-controlled films (22 case index films and 55 control films, including the controls' contralateral and repeat films). Each panel is a Bland–Altman plot of one annotator against the mean of the other two (solid line, bias; dashed lines, 95% limits of agreement). ICC(A,1) = 0.80 (95% CI 0.65–0.88); ICC(A,3) = 0.92 (95% CI 0.85–0.96).

Table S1. Screen of all 135 normalized inter-landmark distances in the primary index-knee cohort (n = 51: 22 revision, 29 control). The table is provided as the Excel workbook Table_S1_all_135_distances.xlsx (one row per distance; columns: combined, AUC and ICC ranks; group means and SDs; difference; Mann–Whitney p; Benjamini–Hochberg FDR-adjusted p; Bonferroni flag; AUC; ICC(A,1) and ICC(A,3) with 95% CIs).

Table S2. Anatomical definitions of the 17 landmarks annotated on the standing anteroposterior radiograph (numbering as in Figure 2). All landmarks were placed on the cortical outline as projected on the AP film.

No.	Landmark	Definition
1	Tibia — lateral plateau	Most lateral point of the lateral tibial plateau margin (subchondral cortex) on the AP projection.
2	Tibia — medial plateau	Most medial point of the medial tibial plateau margin.
3	Tibia — lateral mid-plateau	Midpoint of the lateral tibial plateau articular surface between the lateral margin and the lateral tibial spine.
4	Tibia — medial mid-plateau	Midpoint of the medial tibial plateau articular surface between the medial margin and the medial tibial spine.
5	Tibia — lateral epicondyle	Most prominent point of the lateral tibial metaphyseal flare below the plateau.
6	Tibia — medial epicondyle	Most prominent point of the medial tibial metaphyseal flare below

		the plateau.
7	Tibia — lateral meta-diaphysis	Lateral tibial cortex at the meta-diaphyseal junction, where the metaphyseal flare ends and the diaphyseal cortex becomes parallel.
8	Tibia — medial meta-diaphysis	Medial tibial cortex at the meta-diaphyseal junction.
9	Tibia — lateral shaft	Lateral tibial diaphyseal cortex at the lower border of the annotated field, below landmark 7.
10	Tibia — medial shaft	Medial tibial diaphyseal cortex at the lower border of the annotated field, below landmark 8.
11	Fibula — tip	Apex (styloid) of the fibular head.
12	Fibula — head, lateral cortex	Most lateral point of the fibular head cortex.
13	Fibula — head, medial cortex	Most medial point of the fibular head cortex (adjacent to the proximal tibiofibular joint). Landmarks 12–13 define the fibular head width used for normalization.
14	Fibula — lateral meta-diaphysis	Lateral fibular cortex at the junction of neck and shaft.
15	Fibula — medial meta-diaphysis	Medial fibular cortex at the junction of neck and shaft.
16	Fibula — lateral shaft	Lateral fibular cortex at the level where the shaft attains a parallel cortical outline below the neck. Landmarks 16 and 7 define the lead distance.
17	Fibula — medial shaft	Medial fibular cortex at the same level as landmark 16.
