# Supplementary material for: Fibula–Tibia Distance and Revision for Instability After Primary Total Knee Arthroplasty: An Exploratory Case–Control Study Addressing Rotational Confounding
Source: J Clin Med. 2026 Oct 2;15(19):7658. doi: 10.3390/jcm15197658 (PMC13666231; doi:10.3390/jcm15197658)
Supplement: Supplementary file 1 [file jcm-15-07658-s001.zip › STROBE_Checklist_jcm-4556235.pdf]

STROBE Statement — checklist of items for reports of case–control studies

Manuscript: Fibula–Tibia Distance and Revision for Instability After Primary Total Knee Arthroplasty: An Exploratory Case–Control Study Addressing Rotational Confounding (jcm-4556235, revision 1).

Section references are to the revised (R1) manuscript. Sections: 2.1 Study Design and Population; 2.2 Landmark Annotation and Distance Mapping (including annotation quality control); 2.3 Rotation Sensitivity Analyses; 2.4 Statistical Analysis; 3.1 Cohort; 3.2 Candidate Distance and Internal Validation; 3.3 Inter-Rater Reliability; 3.4 Rotation Sensitivity Analyses; 3.5 Bilateral Agreement; 3.6 Sensitivity Analyses; 4 Discussion and Limitations; 5 Conclusions.

Section / Topic	Item	Recommendation	Where addressed
**Title and abstract**	1a	Indicate the study design with a commonly used term in the title or the abstract	Title: “An Exploratory Case–Control Study”. Abstract, Methods: “exploratory retrospective case–control study”.
	1b	Provide in the abstract an informative and balanced summary of what was done and what was found	Structured abstract (Background/Objectives, Methods, Results, Conclusions) with the analytical unit (index knee), the effect estimate with 95% CI, the internal validation and the rotation sensitivity analyses.
**Introduction — Background/rationale**	2	Explain the scientific background and rationale for the investigation	Section 1, paragraphs 1–2 (the mechanistic link is stated to be hypothetical).
**Introduction — Objectives**	3	State specific objectives, including any prespecified hypotheses	Section 1, paragraph 3 — primary aim (candidate association with revision for instability) and secondary aim (sensitivity to rotation) stated separately.
**Methods — Study design**	4	Present key elements of study design early in the paper	Section 2.1, first paragraph; STROBE cited.
**Methods — Setting**	5	Describe the setting, locations, and relevant dates, including periods of recruitment, exposure, follow-up, and data collection	Section 2.1 — single primary and referral joint-surgery unit; TKA 2014–2024; calendar periods of case and control radiographs stated.
**Methods — Participants**	6a	Give the eligibility criteria, and the sources and methods of case ascertainment and control selection. Give the rationale for the choice of cases and controls	Section 2.1 (“Cases”, “Controls”, “Radiographs and analytical unit”): diagnostic criteria for instability, exclusion of loosening,

			control source and selection, definition of an uncomplicated outcome, follow-up; Figure 1.
	6b	For matched studies, give matching criteria and the number of controls per case	Section 2.1 — frequency matching on age, sex and comorbidity; 30 controls for 24 cases (≈1:1.25); Table 1.
**Methods — Variables**	7	Clearly define all outcomes, exposures, predictors, potential confounders, and effect modifiers. Give diagnostic criteria, if applicable	Sections 2.2–2.4; outcome = revision for instability without loosening; exposure = normalized fibula–tibia distance; rotation proxies and knee side as covariates; Table S2 landmark definitions.
**Methods — Data sources/measurement**	8	For each variable of interest, give sources of data and details of methods of assessment. Describe comparability of assessment methods if there is more than one group	Section 2.2 — identical annotation protocol, three independent blinded annotators, normalization, annotation quality control; Section 3.3 — ICC(A,1) and ICC(A,3) with 95% CIs, Bland–Altman (Figure S2).
**Methods — Bias**	9	Describe any efforts to address potential sources of bias	Section 2.1 (exclusion of malrotated films; index knee in both groups; annotation quality control applied uniformly); Section 2.3 (rotation sensitivity analyses); Section 2.4 (nested internal validation); Discussion paragraph 3 (referral, surveillance and selection bias; calendar period).
**Methods — Study size**	10	Explain how the study size was arrived at	Section 2.1 — all eligible revisions for instability without loosening in the period (n = 24) and 30 matched controls; no formal sample-size calculation (exploratory study); acknowledged in Limitations.
**Methods — Quantitative variables**	11	Explain how quantitative variables were handled in the analyses. If applicable, describe which groupings were chosen and why	Section 2.2 (normalization to fibular head width; alternative scaling in Section 2.4); Section 2.4 — distance analysed continuously (per SD) and at a data-derived

			threshold, explicitly labelled as such.
**Methods — Statistical methods**	12a	Describe all statistical methods, including those used to control for confounding	Section 2.4 — Mann–Whitney U, Bonferroni and FDR, ICC, sum-of-ranks selection, bootstrap optimism correction, nested repeated cross-validation, Hodges–Lehmann and bootstrap CIs, Wilson CIs, logistic regression adjusted for rotation proxies and side.
	12b	Describe any methods used to examine subgroups and interactions	Section 2.4/3.6 — analyses stratified by knee side (Table 4).
	12c	Explain how missing data were addressed	Section 2.4 ("Missing data") — all radiographic measurements complete; the last clinic visit, the primary-arthroplasty date, the age at the annotated radiograph and the documented instability pattern were retrieved from the clinical record for every analysed patient, so Table 1 contains no missing values; the exact primary-arthroplasty date is unavailable for one case operated at another institution and is omitted from the interval summary only.
	12d	If applicable, explain how matching of cases and controls was addressed	Frequency matching on age, sex and comorbidity; balance shown in Table 1 with effect estimates and CIs; not adjusted for matching variables.
	12e	Describe any sensitivity analyses	Sections 2.4 and 3.6, Table 4 — all films without quality-control exclusion; bilateral mean in controls; alternative normalization; unnormalized values; side-stratified; in-silico model across offset and denominator assumptions.
**Results — Participants**	13a	Report numbers of individuals at each stage of the study	Figure 1; Section 3.1.
	13b	Give reasons for non-participation at each stage	Figure 1 — itemised exclusions (187

			revisions for other indications; two annotation quality-control exclusions, and one case whose annotated radiograph postdated the index arthroplasty).
	13c	Consider use of a flow diagram	Figure 1.
**Results — Descriptive data**	14a	Give characteristics of study participants and information on exposures and potential confounders	Table 1 (including follow-up and radiograph calendar year); Section 3.1.
	14b	Indicate the number of participants with missing data for each variable of interest	Table 1 footnote; Section 2.4 ("Missing data").
**Results — Outcome data**	15	Report numbers in each exposure category, or summary measures of exposure	Section 3.2; Table 2; Figure 4A (individual values).
**Results — Main results**	16a	Give unadjusted estimates and, if applicable, confounder-adjusted estimates and their precision. Make clear which confounders were adjusted for and why they were included	Section 3.2 (unadjusted OR at threshold and per SD with 95% CIs); Section 3.4 and Table 3 (OR adjusted for rotation proxies and side); smoking OR with CI in Section 3.1.
	16b	Report category boundaries when continuous variables were categorized	Section 3.2 — data-derived Youden threshold, sensitivity/specificity with Wilson CIs, cross-validated estimates.
	16c	If relevant, consider translating estimates of relative risk into absolute risk for a meaningful time period	Not applicable — case–control design; the Discussion states that odds ratios do not represent absolute or relative risk in unselected patients.
**Results — Other analyses**	17	Report other analyses done — e.g. analyses of subgroups and interactions, and sensitivity analyses	Sections 3.3–3.6; Figures 5–7; Tables 3–4; Table S1.
**Discussion — Key results**	18	Summarise key results with reference to study objectives	Section 4, paragraph 1.
**Discussion — Limitations**	19	Discuss limitations of the study, taking into account sources of potential bias or imprecision. Discuss both direction and magnitude of any potential bias	Section 4, paragraphs 3–4 and Limitations — design, confounding, control selection, calendar period, normalization, rotation model assumptions, laterality effect, annotation quality control.

**Discussion — Interpretation**	20	Give a cautious overall interpretation of results considering objectives, limitations, multiplicity of analyses, results from similar studies, and other relevant evidence	Section 4, paragraphs 1–4; Section 5 — the distance is framed as a hypothesis-generating candidate marker; mechanism untested.
**Discussion — Generalisability**	21	Discuss the generalisability (external validity) of the study results	Section 4, paragraph 3 and Limitations — single-centre, predominantly female cohort; external and three-dimensional validation required.
**Other information — Funding**	22	Give the source of funding and the role of the funders for the present study and, if applicable, for the original study on which the present article is based	Funding statement — this research received no external funding.

An Explanation and Elaboration article discusses each checklist item and gives methodological background and published examples of transparent reporting. The STROBE checklist is best used in conjunction with that article, freely available at https://www.strobe-statement.org.
